# The Emerging Story of Disability Associated with Lymphatic Filariasis: A Critical Review

**DOI:** 10.1371/journal.pntd.0001366

**Published:** 2011-12-27

**Authors:** Lynne Michelle Zeldenryk, Marion Gray, Richard Speare, Susan Gordon, Wayne Melrose

**Affiliations:** School of Public Health Tropical Medicine and Rehabilitation Sciences, James Cook University, Australia; London School of Hygiene & Tropical Medicine, United Kingdom

## Abstract

Globally, 40 million people live with the chronic effects of lymphatic filariasis (LF), making it the second leading cause of disability in the world. Despite this, there is limited research into the experiences of people living with the disease. This review summarises the research on the experiences of people living with LF disability. The review highlights the widespread social stigma and oppressive psychological issues that face most people living with LF-related disability. Physical manifestations of LF make daily activities and participation in community life difficult. The findings confirm the need for the Global Programme to Eliminate Lymphatic Filariasis (GPELF) to support morbidity management activities that address the complex biopsychosocial issues that people living with LF-related disability face.

**Key Learning Points**

1. GPELF indicators continue to be framed around a medical model of health that does not reflect the psychosocial burden of LF. The impact of GPELF interventions on the QOL for people living with LF-related disability remains unknown and unexplored.
2. LF is the leading cause of physical disability in the world. However, there remains a paucity of research from the perspectives of people living with the disease and their perceptions of the impact of current morbidity management programs.
3. Psychological issues and social stigma are experienced by nearly all people living with LF. Interventions to promote psychological well-being and social inclusion should be included in all morbidity management programs.

**Five Key Papers in the Field**

Person B, Addiss D, Bartholomew LK, Meijer C, Pou V, et al. (2008) “Can it be that God does not remember me?” A qualitative study on the psychological distress, suffering, and coping of Dominican women with chronic filarial lymphedema and elephantiasis of the leg. Health Care Women Int 29(4): 349–365.

Perera M, Whitehead M, Molyneux D, Weerasooriya M, Gunatilleke G (2007) Neglected patients with a neglected disease? A qualitative study of lymphatic filariasis. PLoS Negl Trop Dis 1(2): e128. doi: 10.1371/journal.pntd.0000128

Suma T, Shenoy R, Kumaraswami V (2003) A qualitative study of the perceptions, practices and socio-psychological suffering related to chronic brugian filariasis in Kerala, southern India. Ann Trop Med Parasitol 97(8): 839–845.

Ahorlu CK, Dunyo SK, Koram KA, Nkrumah FK, Aagaard-Hansen J, et al. (1999) Lymphatic filariasis related perceptions and practices on the coast of Ghana: implications for prevention and control. Acta Tropica 73(3): 251–261.

Gyapong M, Gyapong J, Weiss M, Tanner M (2000) The burden of hydrocele on men in Northern Ghana. Acta Tropica 77(3): 287–294.

**Research Needs**

1. High quality social science research into morbidity management for LF.
2. Patient perceptions of morbidity management programs. This is urgently needed to identify if current programs meet the needs of patients.
3. Psychological interventions across different stages of the disease that are gender specific.
4. Strategies for culturally appropriate interventions that address social stigma in LF-endemic communities.
5. Interventions that build on existing family and social supports.

## Introduction

Lymphatic filariasis (LF) is a parasitic disease endemic in 81 countries. The global burden is 120 million people, with 40 million people chronically disabled by the disease and about twice that number suffering from covert lymphatic changes or kidney disease [1]. The World Health Organization (WHO) considers LF to be the leading cause of physical disability in the world [2].

LF is caused by three filarial nematodes: *Brugia malayi*, *Brugia timori*, and, most commonly, *Wucheria bancrofti* [3]. These parasites are transmitted via a number of different mosquito hosts, which vary geographically. The most common chronic manifestations of the disease are lymphoedema and hydrocele. Acute adenolymphangitis (ADL), bacterial infections that cause significant pain and fever, also occur in phases. Other less reported clinical expressions include lymphoedema of the breast, swelling of the vulva, and rheumatic and respiratory problems [4].

In 2000, the Global Programme to Eliminate Lymphatic Filariasis (GPELF) was created with the aim of eliminating LF by 2020. This programme is based on two “pillars”: firstly, interrupting the transmission of the parasite by annual, community-wide mass drug administration (MDA), and second, alleviation of suffering of those with chronic manifestations of the disease [5], [6]. The MDA programme is now well advanced with about half of the endemic countries having introduced the strategy [7]. However, morbidity management remains less widespread and successful. Only 26 of the 81 endemic countries have morbidity programs, and most focus only on addressing physical impairment [8]. Outside of Gerusa Dreyer's work [9], there are very few reported programs that support LF patients in actively participating in their communities and achieving greater independence in daily activities.

This paper aims to review the small but important body of qualitative research into the experiences of people living with LF-related disability. Reviews of the social science literature on LF [10] and morbidity management [11] are available; however, no previous review has explored patient reported experiences of disability and management of the condition. Qualitative research is highly valuable, as it attempts to make sense of experiences from participants' lives and the meanings that people bring to them [12]. For the GPELF, high quality qualitative research is vital to inform the second pillar of the programme (alleviating the suffering of those disabled by LF).

### Conceptual Framework of Review

**Figure 1:**
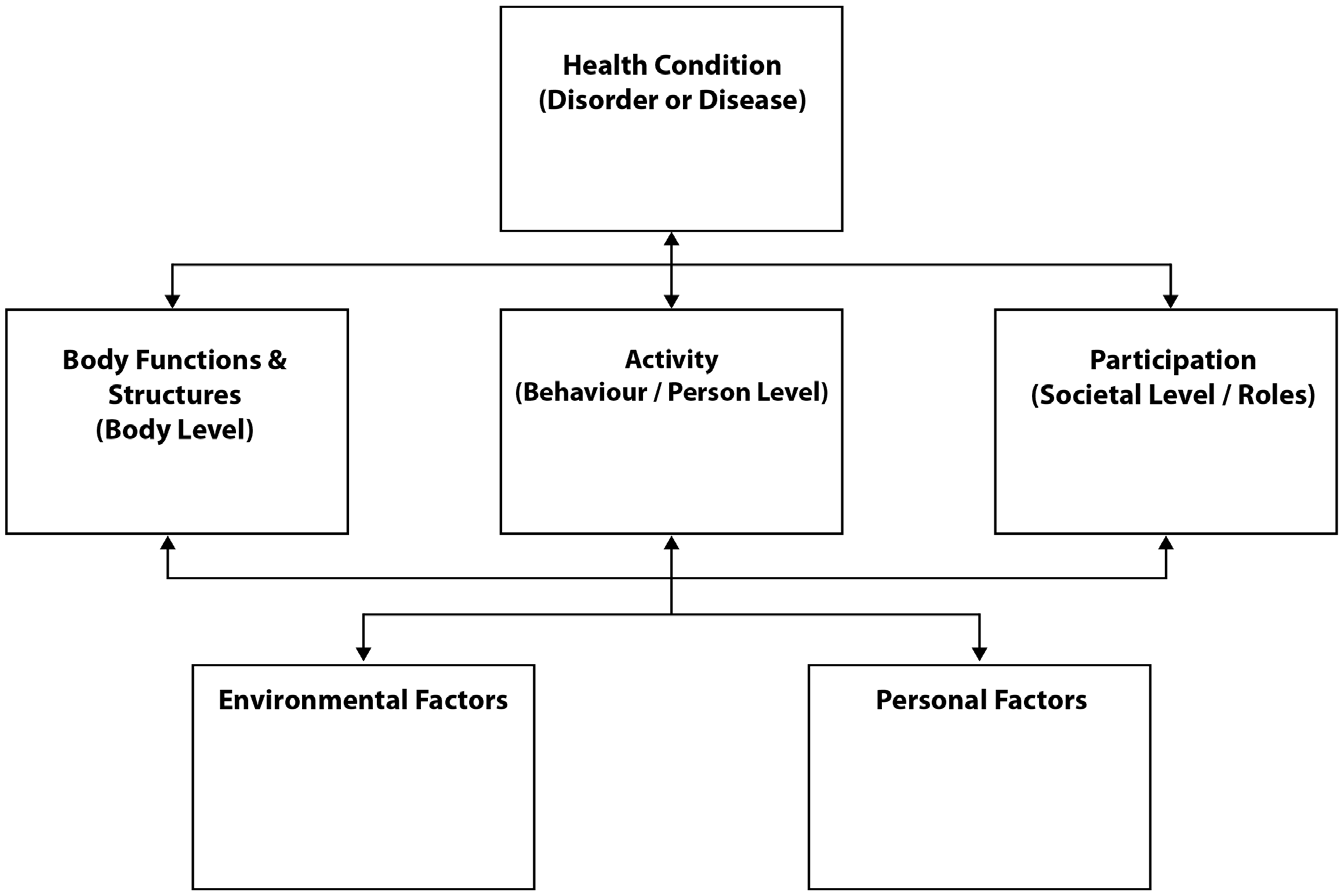
The International Classification of Functioning, Disability and Health (ICF). The ICF model [13] presents a social model of health and functioning that is comprised of six inter-related domains: Health Condition, Body Functions and Structures, Activity, Participation, Environmental Factors, and Personal Factors. Health Condition refers to the presence/absence of a disorder or disease. Body Functions and Structure identifies the impact of physical bodily functioning on health. Within this model, health is seen to be not only the absence of a Health Condition, but also the individual's ability to complete daily Activities of necessity and their Participation in important life roles. Health and functioning is influenced by the existence of Environmental Factors (climactic environment, social attitudes, policies, services, etc.) that can be barriers or supports to health and functioning. Personal Factors (poverty, education level, gender, etc.) also influence health and functioning, depending on the environment a person lives within. Health and functioning is seen within this model as being not only an outcome of a health condition, but also of the other five domains that interact. Hence, health and functioning is seen to depend on context (Environment) and Personal Factors as much as the presence of a health condition and impaired body functions and structures. Finally, impairment is seen not only in terms of reduced bodily functions, but also in terms of a person's inability to complete daily activities and/or to participate in important life roles.

The WHO's International Classification of Functioning, Disability and Health (ICF) is an internationally recognized disability framework [13]. The ICF defines disability as a result of not only disease and impairment, but also of disruptions to one's ability to engage in daily activities and participate in major life roles within their family and community (see Figure 1). The ICF was used as a conceptual framework to guide the search strategy for this review to explore LF patient experiences from a social model of health and functioning. Hence, this review included papers that explored the impact of LF on daily activities and community participation as well as articles that discussed the influence of social, cultural, and institutional impacts.

## Methods

A database search of MEDLINE, CINAHL, Scopus, and ProQuest databases was conducted. Keywords used in the search included “morbidity”, “disability”, “lymphatic filariasis”, “social”, “cultural”, and “quality of life (QOL)”. Manual scanning was also conducted. Articles were included if they 1) were written in English, 2) explored disability issues related to LF, and 3) participants were LF patients and families. Articles were excluded from review if they 1) focused on patient responses to specific treatments, rather than the impact of LF on everyday life, or 2) were quantitative studies. From the database search, 141 articles were identified. One hundred and twenty-two were excluded as they were quantitative in nature, leaving 20 articles for review. Another six articles were excluded as participants were not primarily LF patients.

Articles were critically appraised using the McMaster University Critical Review Form – Qualitative Studies (Version 2.0), which guides systematic review of study purpose, design, sample, data collection and analysis, trustworthiness, conclusions, and implications [14]. This critical review form has previously been used to review literature in other disability studies [15], [16]. A summary of the critical review form is found in Table 1. It is acknowledged that there are limitations to this review. A review of grey literature and articles not published in English could have included more articles for review.

**Table 1:** Summary of Articles Reviewed.

Author & Topic	Design & Sample	Research Aim	Outcomes	Methodological Considerations	Identified Research Needs	Practice Implications
**Ahorlu et al. 2001**Impact of hydrocele on daily lives	*Design not stated*Interviews (*n* = 33); focus groups ×12 (*n* = unstated)	Investigate the impact of hydrocele and hydrocelectomy on pts, their household, and their community.	Hydrocele negatively impacts daily activities, relationships, and social interactions, all of which hydrocelectomy minimises.	Triangulation and cross checking utilised. Minimal consideration of theoretical perspective and researcher bias. Saturation of data not recorded.	Need for further research into hydrocele management practices.	Need for hydrocele management and education within LF morbidity programs. Greater access to low-cost hydrocelectomy has potential to significantly benefit individuals and communities.
**Ahorlu et al. 1999**Health beliefs and perceptions of LF	*Design not stated*Interviews (*n* = 24); focus groups ×5 (*n* = unstated)	Investigate health beliefs and perceptions of LF to inform health education programs.	Elephantiasis seen as shameful and ADL most dreaded issue. Hydrocele not believed linked to other LF presentations.Various misconceptions about cause and management strategies.	Breadth of sample and therefore perspectives included in study. Minimal consideration of theoretical perspective and researcher bias. Saturation of data not recorded.	Investigation into site-specific health beliefs and perceptions necessary for culturally relevant health education programs.	Health education should include and build upon local health perceptions.
**Babu et al. 2009**Impact of hydrocele on sexuality and marriageability	*Ethnography*Focus groups; interviews	To explore impact of hydrocele on sexual functioning and marriageability from pts' and wives' perspectives.	Hydrocele has significant impact on sexual functioning, relationships, and marriageability.Hydrocelectomy known about but not accessed due to cost.	Clear study design, methodological considerations and limitations identified. Minimisation of bias through cross checking and team approach to research. Saturation of data not recorded.	Further research into incidence and impact of hydrocele globally.Research links between MDA and incidence of hydrocele.	Lack of research currently minimises hydrocele as intervention priority. Need for access to low-cost hydrocelectomy to increase uptake.Need for psychological and social interventions for hydrocele.
**Bandyopadhyay 1996**Women and children's experiences and perceptions of LF	*Design not stated*Interviews (*n* = 127)	To investigate the impact of LF for women and children and how they understand and manage the condition Note: aim vaguely stated	Chronic LF has significant impact on productivity, socialisation, relationships.Women less likely to seek treatment. Education at school raised children's understanding of LF.	Methodological approaches vague. Data analysis not described and findings blurred with literature in results. Author's theoretical position and role in research not acknowledged, trustworthiness of research difficult to ascertain.	Further research into gender-specific issues of LF.Further research into genital oedema and impact on sexual functioning and childbirth for women.	Need for women to have greater involvement in control programmes. Community-based approach to health (schools, fairs) essential for women and children to access LF education and management.
**Coreil et al. 1998**Social and behavioural factors in lymphoedema treatment for women	*Design not stated*Interviews (*n* = 24);focus groups ×7 (*n* = 29)	Investigate local understanding of disease, impact of lymphoedema on women's daily lives, and preferred treatment strategies.	Limited understanding of LF aetiology. Lymphoedema limits a range of daily activities (work, self care, care for others, education). Acute attacks leave women reliant on others for care. Need for women to talk with others with condition.	Triangulation of data and a team of experienced researchers increase trustworthiness. Poor description of site of research, limiting transferability of findings. Saturation of data, informed consent, and theoretical perspective of researchers not recorded.	None stated; however, research findings indicate greater research into community perceptions of disease could inform culturally relevant health promotion activities relating to LF.	Need for greater health promotion education on cause and treatment of LF.Women identified strong need for social support programmes, where women can meet others in similar situation as their own and reduce feelings of isolation.
**Gyapong et al. 2000**Men's experience of hydrocele	*Ethnography*Interviews (*n* = 41);focus groups (*n* = unstated)	Investigate the social and economic impact of hydrocele for men with LF.	Hydrocele has negative impact on work, sexual functioning, socialisation, and marriageability.Men desired but feared hydrocelectomy.	Development of methods in consultation with community and triangulation of data are strengths. Minimal discussion of key informants. Saturation of data not recorded.	Greater research into psychological impact of LF associated disability.Research into gender-specific elements and impact of LF disability.	Need for gender-specific health education and interventions for LF morbidity programs.
**Perera et al. 2007**Social and economic impact of filarial elephantiasis	*Design not stated*Interviews (*n* = 60)	Investigate the social and economic impact of filarial elephantiasis from the perspective of those with the disease.	Poverty has great impact on disease progression (delay in seeking treatment, poorer hygiene in the home, occupations that exacerbate condition). Stigma and psychological distress experienced by all, regardless of income.	Clear study design with sampling and auditing process well described. Minimisation of bias through cross checking and team approach to research. Saturation of data not recorded.	Need for LF-specific assessments that classify more than physical impairment.Ongoing need to survey lymphoedema and hydrocele patients within endemic communities to justify expenditure on morbidity management programs.	Need for expansion of lymphoedema treatment programs, informed by local survey statistics.Lymphoedema interventions need to consider social and economic constraints. Need for poverty reduction strategies as part of intervention.
**Person et al. 2006**Health beliefs and self care of lymphoedema	*Grounded Theory*Interviews (*n* = 8);focus group ×3 (*n* = 28)	Investigate the health beliefs, behaviours, and self care of women with lymphoedema.	Cultural practices strongly influenced health-seeking behaviour.All women sought multiple methods for lymphoedema management.	Clear study design with sampling and auditing process well described. Author's theoretical position and role in research not acknowledged, could bias theory development.	Investigation of health beliefs and perceptions necessary to assist planning of culturally relevant health programs.	Family-centred health strategies important for effective lymphoedema management.Training local healers has potential to increase widespread community understanding of LF.
**Person et al. 2007**Social connectedness (SC)	*Grounded Theory*Open ended interviews (*n* = 28);focus groups (*n* = 28)	To identify a) the factors that impact SC and b) the impact of disrupted SC for women with LF.	Age, disease stage, social role, and supports influenced SC. Disrupted SC led to poor health, psychological issues, and social isolation.	Comprehensive data collection and analysis, eliciting clear theory and description of SC. Author's theoretical position and role in research not acknowledged, could bias theory development.	Social science research into the impact on LF on SC.Investigation of the age and stages of LF and relative impact on SC.	Potential need for LF programmes to include social and behavioural interventions in addition to medical approaches.
**Person et al. 2008**Psychological issues and coping strategies of women with LF	*Grounded Theory*Interviews (*n* = 28);focus groups (*n* = 28)	To investigate the psychological state of women with chronic LF and the coping strategies they use.	Shame, depression, social isolation, and hopelessness common for women with all stages of lymphoedema. Self-devised coping strategies helped some women.	Methodological approaches clearly described. Triangulation allowed in depth exploration of issues. Author's role and theoretical position not acknowledged; however, bias minimised with data collection protocols and peer checking.	Development of disability measurement tools that incorporate both physical and psychological impacts of LF (possibly gender specific).	Psychological interventions crucial for LF programs. Use of established cultural supports and LF-specific support groups may be beneficial to maintaining social networks and coping strategies.
**Person et al. 2009**Health-related stigma	*Grounded Theory*Interviews (*n* = 52);focus groups ×6 (*n* = 52)	To investigate the health-related stigma for women living with chronic LF-related lymphoedema.	Women with LF experience enacted, perceived, and internalised health-related stigma. Impact of stigma influenced by social context, personal factors.	Triangulation allowed in depth exploration of issues. Emerging theory well discussed and described. Author's theoretical position and role in research not acknowledged, could bias theory development.	Need for further research into the impact of LF for women, particularly in regard to stigma and QOL. Further research into cross-cultural studies of LF related stigma.	Potential need for LF programmes to include social and behavioural interventions in addition to medical approaches.
**Ramaiah et al. 1997**Functional impairment of LF	*Design not stated*Interviews (*n* = 27);focus groups ×8 (*n* = unstated)	To investigate the functional limitations associated with the different stages of LF.	LF can limit or prevents active engagement in work and domestic tasks.Functional impairment is greatest in acute stages.	Breadth of sample and therefore perspectives included in study. Triangulation of data. Minimal discussion of researcher bias/theoretical framework. Saturation of data not recorded.	Need for reliable and accurate data on LF-related disability to advocate as significant public health issue.Further research into ADL aetiology, impact, and management also required.	None stated. However, results would indicate need for LF programs to incorporate rehabilitation services for to reduce disability and functional impairment caused by LF.
**Suma et al. 2003**Impact and perceptions of chronic disability associated with LF	*Design not stated*Interviews (*n* = 127)	To investigate perceptions and impact of disability on the social lives, marriageability, and productivity of people living with LF.	Later stages of lymphoedema greatly affected work and married life. Stigma greatly felt within community and depression common.	Breadth of sample and therefore perspectives included in study. Minimal discussion of researcher bias/theoretical framework; however, bias minimised by team approach and pre-testing survey. Saturation of data not recorded.	Economic studies into monetary loss associated with LF disability required.Further research into how people can identify early manifestations of disease required to identify and prevent LF disability early.	Health education targeting children required for early intervention.Collaboration with local healers may increase access to LF interventions. Family involvement in disability management may ↑ outcomes. Self-help groups may reduce impact of stigma.

## Findings

### Quality of Evidence

The review highlighted a worrying number of methodological issues within the papers. The majority of papers (seven articles) did not state, describe, or justify their research design, making it difficult to gain a perspective on the frameworks that informed the research. Descriptive clarity was lacking within the majority of articles (eleven articles), with the role and bias of the researchers rarely stated. Sampling was also poorly described by many articles and redundancy in data was often not discussed (eight articles). Analytical rigor in terms of descriptions of analysis and coding techniques was also insufficient in many papers (six articles), although it is acknowledged that limited publishing space makes this difficult. A number of papers (six articles) did not state whether informed consent or ethics approval was gained, highlighting worrying ethical concerns for research within disadvantaged communities.

### Daily Activities

#### Domestic life and self care

The ability to complete household tasks such as cooking, washing, cleaning, and childcare is limited for women with LF-related lymphoedema [17], [18]. Participants reported that acute attacks are particularly debilitating, leaving people dependent on family to assist with the simplest of daily activities such as dressing, bathing, and toileting [19]. In these situations, role reversal can occur, where parents become dependent on their children.

#### Mobility

Physical restrictions due to later stages of lymphoedema and hydrocele decrease people's ability to walk, stand, and sit for long periods and also people's ability to lift heavy loads [17], [19]. Loss of mobility was reported to prevent people from participation in domestic and work roles and was one of the more widely reported causes of lack of income and employment for people living with LF [19]–[21].

#### Sexual relationships

Hydrocele patients state that sexual function is affected by pain, penetration and erection problems, and a reduced desire for sex [22]–[24]. In one study, all hydrocele patients reported decreased sexual functioning, with complications increasing with hydrocele size [22]. Women with lymphoedema who experienced secondary infections also reported avoiding sexual intercourse due to fear of infecting others with LF [18]. Pain during acute attacks was reported to reduce the desire for sex for both genders.

### Participation

#### Interpersonal relationships

The impact of LF on personal relationships and marriageability is significant. Many people marry before physical symptoms become apparent, as visible signs of the disease greatly reduce both men and women's ability to find a partner [23], [25], [26]. In countries where marriage prospects are based on social class, LF patients reported marrying below their class, which negatively impacted the family's social status [23]. Only one study reported that if a girl “has many other good qualities…outshines the other and has charm” then she can still be considered a suitable marriage partner [19].

#### Major life areas

Work roles are routinely interrupted by LF symptoms. Patients with hydrocele report significant difficulties completing work roles, with larger hydrocele preventing any work activity [25], [27]. Hydrocele patients also stated that hard labour, farming, and cultivation jobs worsen their condition [22].

Women with lymphoedema reported adopting a sick role, as they were unable to continue work and other life roles [28]. Women reported that dependence on others dramatically altered, or brought an end to, personally valued social, family, and community roles.

Some children who displayed physical symptoms early in childhood have been reported to be deprived of schooling, as education was believed to be futile [19], [29]. In many communities, it was reported that participation in community events such as church, weddings, funerals, graduations, and community meetings was difficult due to social stigma.

### Environmental Factors

#### Attitudes

The most commonly reported experience reported throughout the literature was social stigma. Participants across many studies reported being publically teased and taunted about physical impairment [29], [30]. Stigma appears to affect members of every social class, and participants believe it is often due to others' misunderstanding of the cause of LF and fear of contagion [19], [28]. Being avoided by others is a common theme for many participants across the studies [18], [27]–[29], [31]. Almost every study described how stigma pervaded all areas of public life for LF sufferers and was felt from families, school and health systems, and within the broader community.

#### Services, systems, and policies

Many participants expressed frustration regarding access to health services. Participants reported prolonged attempts to seek treatment through both local healers and health services, most at considerable expense [32]. Cost of service was reported as a barrier, often leading to ineffective home remedies [21]. Many participants reported ineffective treatments (scarification, bleeding, and amputations) that worsened their condition [24], [32]. In one study, participants who had visited dermatologists trained in simple lymphoedema management were able to describe the cause of lymphoedema and the techniques required to care for their limb [32]. However, most participants within the studies who sought multiple health services did not understand the cause or treatment for LF, indicating that LF awareness is limited within local health services [18], [25]. Participants reported a need for patient support groups and education about the disease (pamphlets, videos, and home visits) [19], [28]. A fear of being identified as an LF patient was reported to prevent many from accessing LF-specific services [21], and many only sought assistance once symptoms significantly impacted work activities [21].

In one study, institutional stigma within the health system was reported where LF patients were being rejected by health services if they had severe physical disfigurement [29]. Institutional stigma was also found to occur within the education system, with reports that schools would not accept some children due to fear of contagion [19], [29].

### Personal Factors

#### Poverty

Throughout the studies, poverty is described as a great personal concern for LF patients. Participants revealed that poverty greatly impedes their ability to seek and access health care [21], [24]. Poorer people were more likely to delay seeking assistance due to the cost of the appointment, treatment, and lost days of work [21]. Even when free medication was provided, the cost of travel and lost time from work prevented people from accessing medication. One study reported that many people with LF live in poorer areas that are more prone to vector breeding grounds and work in unhygienic conditions, placing them at risk of infection and lesions [21].

### Psychological Impact

Without exception, people within the studies reported psychological issues that they directly related to living with their disability. Stress, frustration, and anxiety were commonly reported by participants [17], [26], [29]. In one study, fear and distress was felt strongest in the earlier stages of the disease as the disability began to effect daily life [28].

Poor self-esteem and embarrassment were commonly reported within the studies [18], [19], [28]. Participants reported feeling depressed by their social, economic, and physical situations, which were all worsened by the presence of LF-related disability [18], [29], [33]. Person's studies with women with lymphoedema reveal many women experience a pervading sense of loss of their formal lives [28], [29].

## Discussion

The aim of this paper was to review and critique the qualitative research on the experiences of people living with LF-related disability. Whilst this review has provided useful insight regarding the impact of LF on the daily lives of its sufferers, methodological limitations of the research body is a concern. There is an urgent need for methodologically sound social science research into LF. As with other neglected tropical disease programs, inadequate social research limits the political willingness to implement morbidity management strategies effectively [34], [35]. Whilst MDA programs are being finished in many countries, the story of people living with LF is only beginning to be told.

**Figure 2:**
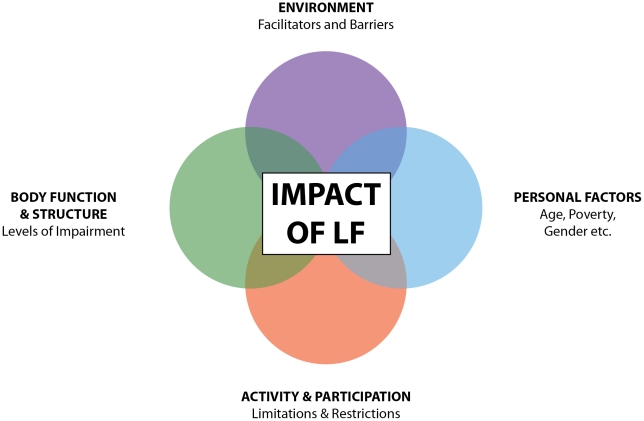
The impact of LF: an adapted ICF model. Figure 2 is an adaptation of the original ICF model [13] outlined in Figure 1. This model shows that the impact of LF is dependent on the interaction of a number of domains within the ICF. The impact of LF is dependent on the impact of the disease on body functions and structures and the level of impairment present. However, the impact of LF is also dependent on the person's environment, which can either facilitate good health (i.e., presence of LF programs within the community) or create a barrier to good health (i.e., the presence of social stigma within the community that prevents people with LF from accessing proper treatment). The impact of LF is seen also in terms of how the disease prevents people from completing daily activities and from participating in major life roles and community events. Finally, LF impact is influenced by personal factors such as poverty, gender, and age. This model shows how these elements (Body Function and Structure, Activity and Participation, Environment and Personal Factors) interact to determine the impact of LF for an individual living with the disease. Hence, LF programs that target solely the body structures and functions level only address one domain and do not respond to other factors that influence the overall impact of LF for a person. Interventions that also address environmental barriers, support re-engagement within daily activities and community participation, and respond to personal needs are theorised to have the greatest impact on the experience of LF for an individual.

This review highlights the complexities of daily life faced by people living with LF-related disability. Conceptualized within the ICF model of health, LF-related disability impairs all domains of function and health, resulting in complex interactions between the environment, body function and structures, personal factors, activity, and participation (see Figure 2). These affect the individual, their family, and their community. It is clear that interventions for LF need to expand beyond those that address only the physical impact of LF and address psychosocial impacts of the disease, a finding that is supported by other studies [36]–[38].

The current GPELF plan states that morbidity management is a priority for 2010–2020, with the following three strategies:

1. Developing guidelines for training modules based on most recent research
2. Developing simple, standardised metrics for morbidity management
3. Improving the integration of lymphoedema care into health systems [8]

The GPELF suggests that programs record the number of people trained in lymphoedema management, number of surgeries performed, and number of patients treated. These reporting priorities continue to reflect a purely medical model of health [39]. Reporting on the number of patients who are actively maintaining self care activities, the number of patients who have seen a reduction in ADL attacks, or the number of patients who had no new disability/had reduced lymphoedema would be more useful outcome measures. Success in increasing patient independence and participation in their communities could also be measured by the number of patient support groups conducted, income generation activities conducted, or patients returning to work/school roles.

The GPELF could learn from the International Federation of Anti-Leprosy Associations (ILEP) Annual Project Report Form, which requires reporting on not only medical interventions, but on the type of rehabilitation programs (self care, support groups, and income generation activities) and number of patients who did not report any new disability [40]. Their programs report yearly on support at the patient (surgery, training, etc.), programme (training, health education, etc.), and rehabilitation (self-help groups) levels [41]. Similar reporting by GPELF would provide evidence of the outcomes within the second pillar globally.

The GPELF's third strategy to integrate greater lymphoedema care within the health system reflects a Western model of health delivery that may not fit within many LF-endemic regions. A focus on lymphoedema management for health services that still do not have basic knowledge on the cause and treatment of LF will not relieve LF patients of the social stigma, psychological burden, activity limitations, and participation restrictions they face, particularly those in later stages of lymphoedema.

A health care model that could be explored for LF is community-based rehabilitation (CBR). CBR is community-driven, low-cost health care that promotes social inclusion for people with disabilities (PWD) [42]. Support given by CBR workers varies; however, it can include exercise and self care education, access to mobility aids, vocational training, support to re-engage in activities of daily living, psychological support, and health promotion activities [43]. Such programs are highly relevant for needs of LF patients found in this review. Whilst there have been examples of the use of community health workers for hygiene management [44], [45] and integrating LF strategies into community programs [46], [47], a strategic approach to engaging with CBR programs for LF management has not been investigated. If the GPELF is planning to develop training manuals, an LF CBR manual similar to that used for leprosy [48] could go a long way to improving local knowledge of the disease and allow LF patients to readily access existing services.

## Conclusions

This paper aimed to review the qualitative literature on the experience of LF-related disability. The findings highlight the complexity of LF-related disability and its impact on the daily activities, participation, and psychological health of people with LF. This review has shown that there is a scarcity of high quality research into LF patient's experiences, indicating that there are many gaps in knowledge remaining. Regardless of the successes of the GPELF programme, millions of people remain burdened by LF-related disability. Whilst there is momentum in the claims of MDA programmes being successful in “eliminating” the disease, millions of people remain impoverished, psychologically damaged, and unable to complete daily activities of necessity. The language that WHO and the GPELF utilize over the coming years is crucial. Claims of widespread “successful elimination programmes” misrepresent the experiences of those already living with LF-related disability and make it increasingly difficult to advocate for morbidity management programmes.
